# Immunoproteomics Analysis of the Murine Antibody Response to Vaccination with an Improved *Francisella tularensis* Live Vaccine Strain (LVS)

**DOI:** 10.1371/journal.pone.0010000

**Published:** 2010-04-02

**Authors:** Susan M. Twine, Mireille D. Petit, Kelly M. Fulton, Robert V. House, J. Wayne Conlan

**Affiliations:** 1 National Research Council Institute for Biological Sciences, Ottawa, Ontario, Canada; 2 DynPort Vaccine Company, LLC, Frederick, Maryland, United States of America; University of Georgia, United States of America

## Abstract

**Background:**

*Francisella tularensis* subspecies *tularensis* is the causative agent of a spectrum of diseases collectively known as tularemia. An attenuated live vaccine strain (LVS) has been shown to be efficacious in humans, but safety concerns have prevented its licensure by the FDA. Recently, *F. tularensis* LVS has been produced under Current Good Manufacturing Practice (CGMP guidelines). Little is known about the immunogenicity of this new vaccine preparation in comparison with extensive studies conducted with laboratory passaged strains of LVS. Thus, the aim of the current work was to evaluate the repertoire of antibodies produced in mouse strains vaccinated with the new LVS vaccine preparation.

**Methodology/Principal Findings:**

In the current study, we used an immunoproteomics approach to examine the repertoire of antibodies induced following successful immunization of BALB/c *versus* unsuccessful vaccination of C57BL/6 mice with the new preparation of *F. tularensis* LVS. Successful vaccination of BALB/c mice elicited antibodies to nine identified proteins that were not recognized by antisera from vaccinated but unprotected C57BL/6 mice. In addition, the CGMP formulation of LVS stimulated a greater repertoire of antibodies following vaccination compared to vaccination with laboratory passaged ATCC LVS strain. A total of 15 immunoreactive proteins were identified in both studies, however, 16 immunoreactive proteins were uniquely reactive with sera from the new formulation of LVS.

**Conclusions/Significance:**

This is the first report characterising the antibody based immune response of the new formulation of LVS in the widely used murine model of tularemia. Using two mouse strains, we show that successfully vaccinated mice can be distinguished from unsuccessfully vaccinated mice based upon the repertoire of antibodies generated. This opens the door towards downselection of antigens for incorporation into tularemia subunit vaccines. In addition, this work also highlights differences in the humoral immune response to vaccination with the commonly used laboratory LVS strain and the new vaccine formulation of LVS.

## Introduction

The facultative intracellular bacterium, *Francisella tularensis*, is pathogenic for many mammalian species including humans, causing a spectrum of diseases collectively called tularemia [1]. Clinically, *F. tularensis* subspecies *holarctica* strains (commonly called type B strains) are responsible for the vast majority of human infections followed by *F. tularensis* subspecies *tularensis* strains (type A strains) [2]. Both subspecies are highly infectious, but only type A strains are able to cause lethal infections in humans [2]. Mortality rates of up to 60% have been reported for untreated human cases of disseminated infection caused by type A strains of the pathogen [3]. In recent years, *F. tularensis* has gained significant attention as one of six organisms designated as high priority agents that could be exploited as agents of bioterror (category A pathogens) by the US Center for Disease Control and Prevention. Combined, the extreme infectivity and ease of dissemination of type A *F. tularensis* have made it a threat to both military personnel and civilians alike.

Currently, there is no licensed vaccine available in the USA to protect against tularemia [4], [5]. A live attenuated strain, designated Live Vaccine Strain (LVS), was derived from a Soviet vaccine strain in the 1960s and is used as an investigational new drug (IND), primarily for the protection of laboratory workers and military personnel. LVS remains the gold standard against which new vaccine candidates are judged. LVS also is the only tularemia vaccine candidate to have been evaluated and shown to be effective in humans. Consequently, there is recent renewed interest in improving the manufacturing and testing of LVS. DynPort Vaccine Company LLC, under contract to the Joint Vaccine Acquisition Program (JVAP) has developed and improved the manufacturing process for *F. tularensis* LVS in compliance with Current Good Manufacturing Practice (CGMP) guidelines. This new vaccine formulation (DVC lot 16 LVS) was the subject of a recent toxicity study in the rabbit [6] and another clinical lot (DVC Lot 17) manufactured using the same process was evaluated in a recent Phase 1 clinical study in humans [7].

In order to license any tularemia vaccine, knowledge of the mechanisms of protection or markers of vaccine ‘take’ will be extremely useful. A series of human trials of LVS, conducted in the 1960s under the name ‘Operation Whitecoat’ demonstrated that most human volunteers vaccinated with LVS were protected against disease symptoms following systemic and aerosol challenge with a virulent type A strain, SCHU S4 [4], and produced agglutinating antibodies to undefined antigen preparations. The identities of the corresponding immunoreactive proteins were not determined, and antibody titers did not predict protection from disease. Ethical considerations prevent a repeat of Operation Whitecoat in the near future, and the natural incidence of tularemia caused by type A *F. tularensis* is too low making it impractical to carry out regular phase 3 clinical trials. Instead, any tularemia vaccine, including LVS, will need to be evaluated for efficacy using the FDA Animal Rule. This will necessitate the development of animal models of tularemia to determine safety, efficacy and correlates of protection.

Previous work has demonstrated that LVS vaccination can protect some mouse strains (e.g., BALB/c, CH3/HeN), but not others (e.g., C57BL/6, DBA) from systemic challenge with type A strains [8]–[10]. Historically, studies in mice successfully vaccinated with LVS have shown that protection against type A strains appears to be mediated predominantly by CD4+ and CD8+ T cells and the cytokine, gamma interferon, rather than by antibodies [11]–[19]. It has been assumed that this is the case also for humans, although more recent work suggests that a combination of cell-mediated and humoral immunity are required for protection [20]. It therefore remains possible that successful vaccination also elicits antigen-specific antibody responses that could potentially serve as independent correlates of protection or markers of vaccine take. Such protein-based markers would be well-adapted to high throughput screening assays that will be used to determine the protection status of individuals post vaccination.

Recently, we and others have used an immunoproteomics approach to determine the repertoire of immunoreactive proteins generated in response to LVS vaccination of mice [21], [22]. These studies showed that mice generate multiple antibody specificities following exposure to *F. tularensis* [21], [22]. These studies used a laboratory strain of LVS and no work has been carried out to characterize the immunoproteomics profile of the new formulation, lot 16 LVS. This current study builds upon our earlier immunoproteomics work, using antisera from BALB/c and C57BL/6 mice immunized with a new formulation of LVS. Thus, the aim of the current work was to evaluate the repertoire of antibodies produced in mouse strains vaccinated with lot 16 LVS.

## Results

Previously, we have shown that *F. tularensis* LVS ATCC 29684 inoculated intradermally elicits a similar sub-lethal infection in the skin, liver, and spleen of both BALB/c and C57BL/6 mice that persists for approximately 2 weeks [8]. However, whereas this infection renders BALB/c mice immune to a subsequent systemic challenge with >100 LD_50_ of a virulent type A strain of *F. tularensis*, it fails to protect C57BL/6 mice from a 100-fold smaller challenge [8]. In an earlier study, we used an immunoproteomics approach to determine whether protective immunity correlated to a difference in specific antibody response [23]. The current study builds upon this work, using antisera from BALB/c and C57BL/6 mice immunized with a new formulation of LVS (lot 16 LVS).

**Figure 1:**
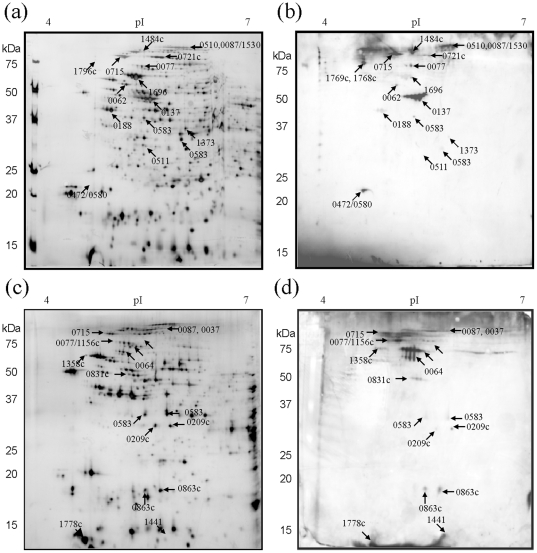
Two-dimensional immunoblots of *Francisella tularensis* LVS protein extracts probed with sera from BALB/c mice successfully vaccinated with DVC-LVS Lot16. (a) Representative silver stained reference 2D-PAGE of LVS total protein lysates separated in pH range 4–7 and b) equivalent immunoblot. (c) LVS membrane enriched fractions, separated in pH range 4–7 and (d) corresponding immunoblot. Immunoreactive areas are labeled on Western blot images and the corresponding immunoreactive proteins are indicated on silver stained gels in (a) and (c). The annotation numbers indicate the protein locus tag and are summarized in Table 1.

**Figure 2:**
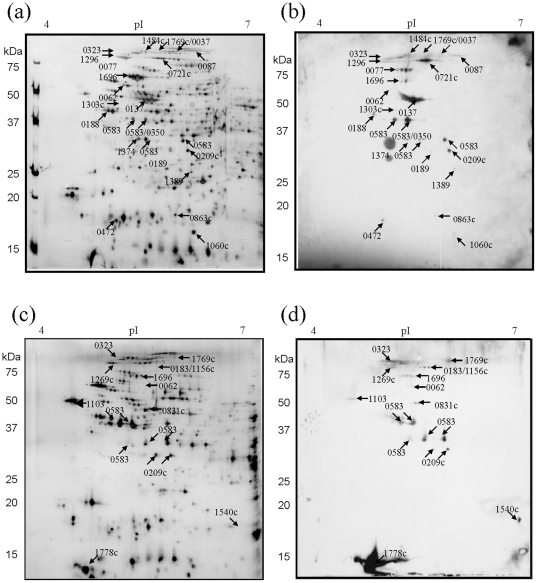
Two-dimensional immunoblots of *Francisella tularensis* LVS protein extracts hybridized with sera from C57BL/6 mice unsuccessfully vaccinated with DVC-LVS Lot16. (a) Representative silver stained reference 2D-PAGE of LVS total protein lysates separated in pH range 4–7 and b) equivalent immunoblot. (c) LVS membrane enriched fractions, separated in pH range 4–7 and (d) corresponding immunoblot. Immunoreactive areas are labelled on Western blot images and the corresponding immunoreactive proteins are indicated on silver stained gels in (a) and (c). The annotation numbers indicate the protein locus tag and are summarized in Table 1. Two spurious areas on blot (d) were observed and did not correspond to areas of immunoreactivity.

Our experimental strategy used 2D-Western blotting of total soluble protein and membrane protein enriched fraction of LVS lot 16, with proteins resolved across three partially overlapping pH ranges. No immunoreactive proteins were observed with pI values of less than 4 or greater than 7, therefore the data presented here are limited to the separation range between pH 4–7. All sera were used at appropriate dilutions, based on our previous work [23] and no reactions with naïve control sera were observed. To examine the *Francisella-*specific antibody response, all immunoblots were performed in duplicate and representative blots are shown herein (Figures 1 and 2).

### Profile of immunoreactive proteins with antisera generated by successful vaccination of BALB/c mice with lot 16 LVS

Figure 1b shows 2D-Western blots of the lot 16 LVS total soluble protein extract, separated between pH 4–7, probed with antisera pooled from five lot 16 LVS vaccinated BALB/c mice. Twenty-eight individual areas of immunoreactivity of varying intensity were observed. All areas of immunoreactivity were aligned to protein spots on equivalent silver stained 2D reference gels (Figure 1b) and these protein spots were subsequently identified using nano-LC MS/MS. In other cases, for example FTT_0137, elongation factor Tu, was identified as a large immunoreactive area, that corresponded to a ‘spot train’ on the corresponding protein stained 2D gel (Figure 1a). In such cases where all immunoreactive areas within a spot train were identified as the same protein, a single arrow was used to identify the protein in Figure 1a & b. The identified proteins are summarized in Table 1 (specific details of peptide MS/MS scores shown in Table S1) with a total of 18 unique proteins identified as reactive with sera from lot 16 LVS immunized BALB/c mice. These areas of immunoreactivity and protein spots are indicated in Figure 1. In some cases, the same immunoreactive protein focused to more than one area on a gel, with protein spots differing slightly in isoelectric point. For example, the membrane protein FTT_0583 was observed to be immunoreactive in two discrete protein spots, differing in both MW and pI (Figure 1a & b).

**Table 1:** *Francisella tularensis* live vaccine strain immunoreactive proteins.

LocusTag (a)	Protein Name	Experimental localization(b)	Observed Immunoreactivity (this study) (c)		PSORT, COG (d)	Previously observed(e)
			BALB/c	C57BL/6		
**Proteins immunoreactive with sera from successfully vaccinated mice only**						
FTT0064	ATP synthase beta chain	M	**+++**		Cytoplasmic,C	
FTT0510	DNA gyrase subunit B	TP	+++		Cytoplasmic,L	
FTT0511	Pyridoxine/pyridoxal 5-phosphate biosynthesis	TP	+		Cytoplasmic, L	
FTT0580	Hypothetical protein	TP	++		Cytoplasmic, R	
FTT0715	Chitinase family 18 protein	TP, M	**+++**		Unknown, G	[22], [23] M
FTT1358c	Intracellular growth locus subunit B	M	+		Unknown, S	
FTT1373	3-oxoacyl-[acyl carrier protein] synthase III	TP	+		Unknown, I	
FTT1441	Hypothetical protein	M	++		Cytoplasmic, P	[24] H
FTT1530	Fusion product of 3-hydroxacyl-CoA	TP	+++		Cytoplasmic, I	
**Proteins immunoreactive with sera from successfully and unsuccessfully vaccinated mice**						
FTT0037	NADH dehydrogenase I G subunit	M	++	+	Unknown, C	
FTT0062	ATP synthase alpha chain	TP	+	+/−	Unknown, C	
FTT0077	Dihydrolipoamide succinyltransferase	TP, M	+++	++	Cytoplasmic, C	[23], [24], MH
FTT0087	Aconitate hydratase	TP, M	+	+	Cytoplasmic, C	[24,24] H
FTT0137	Elongation factor Tu (EF-Tu)	TP	++++	+	Cytoplasmic, J	[22], [23] M
FTT0188	Cell division protein	TP	+	+	Cytoplasmic, D	[23], [24] MH
FTT0472	Acetyl-CoA carboxylase biotin carboxyl carrier	TP	+/−	+	Unknown, I	[22], [23] M
FTT0583	Outer membrane associated protein	TP, M	+ *_+_	++	OM, M	[21]–[23] MH
FTT0721c	Peroxidase/catalase	TP	+	+++	OM, P	[22]–[24] MH
FTT0831c	OmpA family protein	M	++	++	Unknown, M	[24] H
FTT0863c	LemA-like protein	TP M	++	+	Cytoplasmic, S	[23], [24] MH
FTT0209c	Periplasmic solute binding protein	M	+	++	Unknown, P	
FTT1156c	Type IV pilin multimeric outer membrane protein	M	++	++	OM, U	
FTT1484c	Pyruvate dehydrogenase E2 component	TP	++	+	Cytoplasmic, C	[24] H
FTT1696	Chaperonin GroEL	TP, M	++++	++++	Cytoplasmic, O	[22]–[24] MH
FTT1769c	ClpB protein	TP, M	+	+	Cytoplasmic, O	[24] H
FTT1778c	Hypothetical membrane protein	M	+	+++	Unknown, -	[23] M
**Proteins immunoreactive with sera from unsuccessfully vaccinated mice only**						
FTT0183c	30S ribosomal protein S1	M		++	Cytoplasmic, J	[23] M
FTT0189	UDP-3-O-[3-hydroxymyristoyl]	TP		+	Unknown, M	
FTT0323	Elongation factor G (EF-G)	TP		++	Cytoplasmic, J	[23] M
FTT0350	DNA-directed RNA polymerase alpha subunit	TP		++	Cytoplasmic, K	
FTT1060c	50S ribosomal protein L9	TP		+/−	Cytoplasmic, J	
FTT1103	Conserved hypothetical lipoprotein	M		+	Unknown, O	[23], [24] MH
FTT1303c	Hypothetical protein	TP		+	Unknown, -	
FTT1374	Malonyl coA-acyl carrier protein	TP		+	Cytoplasmic, I	
FTT1389	3-methyl-2-oxobutanoatehydroxymethyltransferase	TP		+	Unknown, H	
FTT1540c	Hypothetical protein	M		++	Unknown, R	
FTT1269c	Chaperone protein	TP, M		+	Periplasmic, O	[21]–[23] MH

(a) Locus tag from SCHU S4 database. This corresponds to numerically annotated immunoreactive areas in Figure 1.

(b) Indicates whether protein reactivity was observed in DVC LVS total soluble proteome extract (TP) or membrane enriched proteome (M).

(c) Indicates whether immunoreactivity was observed towards each protein was observed with immune sera from LVS vaccinated BALB/c or C57BL/6 mouse strains. From–(no reactivity), (+/−) at the limits of detection, to ++++ (intense reactivity).

(d) PSORT–predicted subcellular location. COG- Clusters of Orthologous groups, functional annotation based upon protein sequence. OM indicates ‘outer membrane’.

(e) Number indicates reference in which protein immunoreactivity was previously reported. ‘M’ or ‘H’ indicate whether the reported study used sera drawn from murine models of tularemia (M) or human subjects (H).

*Total intensity for all immunoreactive areas identified as FopA. Details of scoring for protein identification by using tandem mass spectrometry are shown in supplementary table S1.

2D-Western blots, using the membrane enriched lot 16 LVS proteome as the antigen, were also probed with sera from lot 16 LVS immunized BALB/c mice. As shown in Figure 1d, this blot showed 16 distinct areas of immunoreactivity. Subsequent protein identification using mass spectrometry gave rise to 15 unique proteins (Table 1). Eight of the immunoreactive proteins were only detected in the membrane enriched proteome fraction (ATP synthase beta chain (FTT0064), Intracellular growth locus subunit B (FTT1358c), Hypothetical protein (FTT1441), NADH dehydrogenase I G subunit (FTT0037), OmpA Family Protein (FTT0831c), Periplasmic solute binging protein (FTT0209c), TypeIV pilin multimeric outer membrane protein (FTT1156c), and hypothetical protein (FTT1778c)). By contrast, seven proteins were identified in both membrane enriched and total soluble proteome fractions. These proteins were ClpB protein (FTT1769c), Chitinase family 18 protein (FTT0715), Outer membrane associated protein FopA (FTT0583), Chaperonin GroEL (FTT1696), LemA-like protein (FTT0863c), Dihydrolipoamide succunyl transferase component of 2-oxoglutarate dehydrogenase complex (FTT0077), and Acontitate hydratase (FTT0087).

When considering all the proteins immunoreactive with immune sera from LVS vaccinated BALB/c mice, 15 had previously been reported in the literature as immunoreactive with either murine or human sera (indicated in Table 1) [21], [23], [24]. Eleven of the identified immunoreactive proteins have not, to our knowledge, previously been documented to be immunoreactive with *Francisella* antisera, including intracellular growth locus subunit B (FTT1358c), ATPsynthase beta chain (FTT0064), type IV pilin multimeric outer membrane protein (FTT1156c) and NADH dehydrogenase G subunit (FTT0037).

### Profile of immunoreactive proteins with antisera generated by unsuccessful vaccination of C57BL/6 mice with lot 16 LVS

2D-Western blots of lot 16 LVS total soluble proteome were probed with pooled antisera from 5 LVS vaccinated C57BL/6 resulting in a total of 40 areas of immunoreactivity (Figure 2b). Alignment of blots with equivalent protein stained 2D-PAGE gels allowed identification of 20 unique proteins (Figure 2b & d and Table 1, with specific details of peptide MS/MS scores shown in Supplementary Table S1). This is in contrast to our previous immunoproteomics study using antisera from C57BL/6 mice vaccinated with a laboratory passaged strain of LVS, which resulted in the identification of only four intensely reacting protein spots within the LVS proteome [23].

Western blots of the lot 16 LVS membrane enriched proteome showed 16 areas of immunoreactivity when probed with antisera from vaccinated C57BL/6 mice (Figure 2d). All but one area of immunoreactivity was aligned with corresponding silver stained protein gels and 15 unique proteins were identified (Table 1). Certain proteins, such as the outer membrane protein, FopA and the periplasmic solute binding protein were found to focus in several distinct immunoreactive isoforms on 2D-PAGE, which differed markedly in isoelectric point (Figure 2c). This may represent genuine isoforms of the protein, and was consistently observed on all protein stained 2D gels. The immunoreactivity of each protein isoform, however, varied with the sera used to probe the blot. Seven immunoreactive proteins were detected in both total proteome and membrane enriched fractions, while eight were uniquely identified in the membrane enriched fraction.

When considering the total 28 proteins that were immunoreactive with sera from C57BL/6 vaccinated mice, eleven of these proteins were observed to be reactive only with sera from immunized mice from this strain. The remaining 17 proteins were also immunoreactive with sera from LVS vaccinated BALB/mice. In contrast, 9 of the 28 proteins observed to be immunoreactive with sera from BALB/c vaccinated mice were not immunoreactive with sera from vaccinated C57BL/6 mice.

### Functional classification of immunoreactive proteins and potential diagnostic markers of vaccination

The immunoreactive proteins were classified according to their computationally predicted features. These data are summarized shown in Table 1. The PSORTb algorithm (http://www.psort.org/psortb/) predicts protein subcellular location for Gram negative bacteria, based upon protein sequence. This was used to predict the subcellular location of the immunoreactive proteins identified in this work and showed cytoplasmic proteins to be enriched, consisting of 50% of the total identified immunoreactive proteins. Thirty-eight percent of the immunoreactive proteins could not be predicted to localize to a specific subcellular location. Twenty-nine percent of this subset of immunoreactive proteins were hypothetical proteins. Overall, the antigenic proteins were derived from diverse functional categories, including chaperonin proteins, protein synthesis and carbohydrate metabolism. Both chaperonins and proteins involved in aspects of energy metabolism were highly represented among the immunoreactive proteins. Stress response proteins have previously been reported to react with convalescent sera in both tularemia and other diseases [21], [24]–[26]. The immunoreactive proteins included several proteins that were observed to be increased in expression during the later stages of murine tularemia (Acetyl CoA caboxylase, Chitinase family 18 protein, Peroxidase/Catalase and hypothetical protein FTT1303c) [27].

A total of nine proteins, combined from total protein and membrane fraction, were observed only to be reactive with sera from lot 16 LVS vaccinated BALB/c mice (ATP synthase beta chain, DNA gyrase subunit B, Pyridoxine/pyridoxal 5-phosphate biosynthesis protein, Hypothetical protein FTT0580, Intracellular growth locus subunit B, 3-oxoacyl-[acyl carrier protein] synthase III, Hypothetical protein FTT1441 and Fusion product of 3-hydroxacyl-CoA Dehydrogenase and acyl-CoA-binding protein). These nine proteins represent the first stage in the identification of antibody-based markers of successful vaccination. By contrast, 11 of the protein spots were found to be immunoreactive to antisera from both BALB/c and C57BL/6 vaccinated mouse strains (Table 1). A further 11 proteins were reactive only with sera from DVC LVS vaccinated C57BL/6 mice.

## Discussion

There is a need for a safe and effective tularemia vaccine to address potential bioterrorism threats. Historically, tularemia live vaccines were successfully used in the former Soviet Union to protect the general population against type B endemics, and in the West to protect tularemia researchers against type A bacteria [5], [28], [29]. In human volunteer studies conducted more than 40 years ago, most vaccinees immunized with *F. tularensis* LVS were protected against subsequent pulmonary or systemic exposure to a highly virulent type A strain of the pathogen [30]–[32]. However, 10–30% of vaccinees remained vulnerable to such challenge despite seroconversion to undefined *Francisella* antigens [33]. No correlation between the agglutinating antibody titre to these antigens and level of protection against virulent *F. tularensis* was found [30], [31], [31]–[33]. Identification of correlates of protection will undoubtedly aid efforts to license any potential tularemia vaccine. At present, LVS remains the only vaccine candidate to show efficacy in humans. When testing a vaccine in human clinical trials is impossible, the U.S. Food and Drug Administration offers an alternative path to vaccine licensure using the so-called “Animal Rule”, whereby the efficacy of such vaccines can be demonstrated through animal studies. Application of the Animal Rule to current and future tularemia vaccine candidates would be facilitated if an immunological correlate of protection or marker of vaccine take in an animal model was identified in order to bridge efficacy in animals to immunogenicity in humans. The lot 16 LVS preparation was produced under CGMP guidelines (described in [6]) and is currently being characterized for safety in various animal models and in humans [6], [34]. For example, preliminary safety and immunogenicity of the lot 16 LVS was conducted in rabbits [6], but a concomitant challenge study was not performed. The murine model of tularemia is well characterized in terms of pathogenesis and immune response (reviewed in [35]) and represents an accessible animal model for defining the immunogenicity of the lot 16 LVS formulation. By comparing the repertoire of immunoreactive proteins generated by successful and unsuccessful vaccination of mice with LVS, we have gained insight into immunoreactive proteins that may serve as markers of successful vaccination. BALB/c mice, successfully vaccinated with lot 16 LVS, generated antibodies towards nine proteins that were not recognized by sera from unsuccessfully vaccinated C57BL/6 mice.

A comparison of the murine immunoreactive proteins identified in our current work with those identified in our earlier study, where mice were vaccinated with a laboratory strain of LVS, shows some overlap and some noticeable differences. A total of 15 immunoreactive proteins were identified in both studies, indicating commonalities in the immune response to LVS strains derived from different sources. However, 16 immunoreactive proteins identified in this study, were not identified in our previous work. The most notable difference was observed in the immunoproteomic profiles of sera from LVS vaccinated C57BL/6 mice. Previously, sera from C57BL/6 mice vaccinated with laboratory LVS showed limited reactivity towards a small number of proteins. By contrast, the newer lot of LVS preparation appeared to stimulate a larger repertoire of antibodies in C57BL/6 mice even though it still did not confer protection against subsequent challenge. The reasons for the observed differences in antigenic protein profiles remain unknown. Alternatively, the differences may stem from the origins and preparations of the bacteria, such as improvements in vaccine manufacture. Minor differences between the laboratory passaged LVS and the newer LVS preparation have been observed at the proteome level (unpublished data) and it remains to be determined whether this has contributed to the observed differences between the two studies.

The most challenging task in tularemia research, including the development of correlates of protection, is demonstrating that the findings in animal models are applicable to humans. Literature reports of studies of the immunoproteome of LVS (ATCC29684) with sera from tularemia patients have been added to a summary of the data from the current study (Table 1). Little information regarding the source of the human sera, the time after infection and the protected status of the subjects are known. However, 13 proteins reported to be immunoreactive to human *Francisella* antiserum [21], [24] are also observed to be immunoreactive to murine antisera in this current study. Of those proteins only two were found exclusively to react with sera from successfully vaccinated mice. These proteins were identified as OmpA and hypothetical protein FTT1441. Whilst it is not feasible to determine whether the immunoreactivity to these proteins is indicative of the protected state of the human host, these proteins represent leads in the search for protein based markers of vaccine take for LVS vaccination. In this regard, immunoproteomics studies of sera from other animal models of tularemia using different host species, or human clinical trials will provide additional information regarding antigens that are immunoreactive across various species. It will then be possible to down-select to commonly reactive protein antigens that can be incorporated into an assay to rapidly screen sera for the presence of antibody markers of successful vaccination. These data will also be useful in down selecting to antigens that might be used in a protein based subunit vaccine.

## Materials and Methods

### Bacteria

The LVS strain used in murine immunizations was derived directly from a vial of DVC lot 16 LVS (DVC Lot #703-0303-016). This new formulation was derived from LVS NDBR101 lot 4, the history of which is briefly described elsewhere [6] and has been produced using standardized fermentation, purification and formulation processes. Working bacterial stocks were prepared as described elsewhere [8], [9].

### Murine vaccine sera

Mouse challenge experiments were approved by and performed at the National Research Council of Canada, Institute for Biological Sciences in a federally-licensed small animal containment level 3 facility that is also approved by the NIH for Select Agent research. Specific-pathogen-free female BALB/c mice were purchased from Charles River Laboratories (St. Constant, Que.). Mice were maintained and used in accordance with the recommendations of the Canadian Council on Animal Care Guide to the Care and Use of Experimental Animals. For intradermal inoculations, stocks of the strains were diluted in sterile saline. Actual concentrations of inocula were determined by plating. Intradermal inocula (50 µl/mouse) were injected into a fold of skin in the shaved mid-belly.

BALB/c (n = 5) and C57BL/6 (n = 5) mice were immunized intradermally (ID) with ∼5×10^4^ colony forming units of reconstituted LVS lot 16. Mice were bled 28 days post-vaccination and pooled sera were used to probe 2D Western blots of LVS antigens. When these same mice were challenged intradermally 53 days post-vaccination with 1000 LD50 of the fully virulent SCHU S4 strain, 5/5 C57BL/6 mice died between days 6–9, whereas 5/5 BALB/c mice survived to 20 days without any overt signs of infection.

### Two-dimensional polyacrylamide gel electrophoresis (2D PAGE) and immunoblot analysis

Proteins were extracted either using a one-step extraction procedure as described previously, to form total soluble protein extracts [36]. Briefly, bacteria were grown in modified Mueller-Hinton broth for 24–36 h at 37°C with shaking until bacterial density reached 10^8^–10^10^ CFU/mL. Bacteria (LVS) were grown, harvested and lysed within a BioSafety (BS) Level 2 containment facility. Bacterial cultures were harvested in 1 mL aliquots by centrifugation and the pellets were washed three times with sterile, distilled water. Cell pellets were then resuspended in twelve times the pellet volume of lysis solution (5 M urea, 2 M thiourea, 1% DTT, 4% CHAPS, 0.5% ASB-14).

Crude membrane protein extracts were prepared as described previously [36], [37]. Bacterial cells were harvested from broth culture to give a final pellet containing ∼10^10^ bacteria. The pellet was washed twice with distilled water before resuspending in 4 mL of 50 mM Tris/HCL, pH 7.3 with 0.7 mg DNase I (Sigma). The cells were disrupted by sonication and unbroken cells removed by centrifugation at 2500×g for 10 min. The supernatant was diluted to a final volume of 50 mL with ice-cold 0.1 M sodium carbonate, pH 11 and the solution was gently stirred at 4°C for 1 h. Carbonate treated membranes were collected by ultracentrifugation in a Beckman 55.2 Ti rotor at 100 000×g for 1 h at 4°C. The supernatant was discarded and membrane pellet resuspended in 5 mL of ice cold 50 mM Tris/HCl to remove contaminants, and then collected by centrifugation at 100 000×g for 30 min. This wash procedure was repeated a second time, again discarding the supernatant. The final membrane protein containing pellet was solubilized for 2D electrophoresis in 1.0 mL of IEF solution (7 M urea, 2 M thiourea, 1% (w/v) ASB-14, 4% (w/v) CHAPS, 1% (w/v) DTT, and 0.5% (v/v) Biolytes 3–10 (Bio-Rad, Mississauga, ON).

The extracted proteins were separated using immobilized pH gradient strips (IPG), either linear pH 3–6, 4–7, 17 cm (Bio-Rad, Mississauga, ON) or linear pH 6–11, 18 cm (GE Healthcare, Baie d'Urfe, QC) essentially as described previously [23] using 100 µg of protein/gel. Second dimension PAGE gels were run in duplicate, with the first used for immunoblotting and the second silver stained to serve as a 2D reference map for protein spot identification.

Immunoblotting was carried out according to methods previously published by others [*38*]. Proteins separated by 2D PAGE were electroblotted onto PVDF membranes (Bio-Rad, Mississauga, ON) at 15 V for 1 h using a semi-dry Trans Blot Cell (Bio-Rad, Mississauga, ON). PDVF membranes were incubated overnight in phosphate-buffered saline/Tween (PBST; (9 mM sodium phosphate, 0.15 M NaCl, and 0.05% v/v Tween 20) containing 5% w/v skim milk powder at 4°C with constant rotation. Following two 5 min washes with PBST, the PVDF membranes were re-incubated with mouse anti-*Francisella* serum; anti-LVS sera were diluted 1∶1000 in PBST containing 5% w/v skim milk powder. Incubation was for 1 h at room temperature with constant rotation. After washing with PBST, blots were then incubated with peroxidase-conjugated goat anti-mouse immunoglobulin (Perkin-Elmer Life and Analytical Sciences, Woodbridge, Ontario). This was diluted 1∶5000 in PBST containing 5% w/v skim milk powder. Incubation was for 1 h at room temperature. Reactive spots were visualized using the Western Lightning Chemiluminescence kit (Perkin-Elmer Life and Analytical Sciences, Woodbridge, Ontario) and images captured/transferred onto BioMax Film (Perkin-Elmer Life and Analytical Sciences, Woodbridge, Ontario). Immunoblotting experiments were conducted in duplicate, with no variation in results observed. Images of immunoblots were captured using FluorS Scanner (Bio-Rad, Mississauga, ON) and aligned with equivalent protein stained 2D gels using PDQuest software (Bio-Rad, Mississauga, ON).

### In-gel digestion and protein identification

Selected protein spots were excised and tryptically digested as described previously [23]. Briefly, spots were excised manually from silver stained 2D-PAGE and destained with 15 mM potassium hexacyanoferrate, 50 mM sodium thiosulfate. Protein spots were digested with 10 ng/uL trypsin in 50 mM ammonium bicarbonate at 37°C for 16 hours. The resulting peptides were analysed by nano-electrospray tandem mass spectrometry (nLC-MS/MS). With a flow rate of 0.4 uL/min, peptides were eluted from a 100 µm i.d. ×100 mm nanoAcquity UPLC 1.7 µm C_18_ column (Waters, Mississauga, Ontario) with the following gradient: 1% B for 1 minute, 1%–45% B over 18 minutes, 45%–85% B over 3 mninutes, 85%–1% B over 1 minute. The column was re-equilibrated with 1% B for an additional 8 minutes. Solvent A is 0.1% formic acid in Optima LCMS water (Fisher Scientific Canada, Whitby, Ontario). Solvent B is 0.1% formic acid in acetonitrile. The peaklist files of MS^2^ spectra of the excised protein spots were searched against a database (2008.03.10) with 11947 entries consisting of the NCBI reference genomes for 7 strains of *Francisella* (NCBI ids: NC_006570, NC_007880, NC_008245, NC_008369, NC_008601, NC_009257, NC_009749) using the MASCOT™ search engine (version 2.2.03) (Matrix Science) for protein identification. The mass tolerance used for precursor ions was ±0.8 Da and the mass tolerance for fragment ions was ±0.15 Da. One missed cleavage site was permitted. The cut-off ions score was 30, above which ion scores indicate identity. In addition, all spectral matches were verified manually.

## Supporting Information

Table S1 nLCMS/MS identification of immunoreactive proteins from tryptic digests of protein gel spots.
(0.09 MB DOC)
