# Supplementary material for: Immunoproteomics Analysis of the Murine Antibody Response to Vaccination with an Improved Francisella tularensis Live Vaccine Strain (LVS)
Source: PLoS One. 2010 Apr 2;5(4):e10000. doi: 10.1371/journal.pone.0010000 (PMC2848853; doi:10.1371/journal.pone.0010000)
Supplement: Table S1 — nLCMS/MS identification of immunoreactive proteins from tryptic digests of protein gel spots. (0.09 MB DOC) [file pone.0010000.s001.doc]

| **Locus Tag ^a^** ) | **MW, p*I***  **^b)^** | **Mascot Score ^c)^** | **Sequence Coverage ^d)^** | **Protein Informatione^e^**) | |
| --- | --- | --- | --- | --- | --- |
|  |  |  |  | **Definition** | **Accession No.** |
| FTT0062 | 55.4, 5.02 | 331 | 20 | ATP synthase alpha chain | *YP_169137* |
| FTT0064 | 49.8, 5.07 | 397 | 40 | ATP synthase beta chain | *YP_169139* |
| FTT0077 | 52.7, 5.13 | 2065 | 46 | Dihydrolipoamide succinyltransferase | YP_169152 |
| FTT0087 | 102.6, 5.44 | 453 | 20 | Aconitate hydratase | YP_169161 |
| FTT0137 | 43.4, 5.12 | 14961 | 77 | Elongation factor Tu (EF-Tu) | YP_169203 |
| FTT0183 | 61.5, 5.16 | 361 | 24 | 30S ribosomal protein S1 | *YP_169244* |
| FTT0188 | 39.7, 4.76 | 19031 | 89 | Cell division protein | YP_169249 |
| FTT0189 | 32.0,5.27 | 242 | 21 | acetylglucosamine deacetylase | YP_169250 |
| FTT0209c | 33.7, 5.46 | 86 | 21 | Periplasmic solute binding family protein | *YP_169268* |
| FTT0323 | 77.6, 4.97 | 916 | 32 | Elongation factor G (EF-G) | YP_169372 |
| FTT0350 | 35.3, 4.93 | 1623 | 70 | DNA-directed RNA polymerase | YP_169399 |
| FTT0472 | 16.5, 5.00 | 3946 | 85 | Acetyl-CoA carboxylase, biotin carboxyl | YP_169510 |
| FTT0510 | 90.0, 5.41 | 1114 | 46 | DNA gyrase subunit B | YP_169545 |
| FTT0511 | 30.8, 5.18 | 131 | 46 | Pyridoxine/pyridoxal 5-phosphate | YP_169546 |
| FTT0580 | 20.3, 4.57 | 119 | 55 | Hypothetical protein | YP_169604 |
| FTT0583 | 41.4, 5.58 | 141 | 13 | Outer membrane associated protein | YP_169607 |
| FTT0583 | 41.4, 5.58 | 416 | 41 | Outer membrane associated protein | *YP_169607* |
| FTT0715 | 83.0, 4.96 | 4333 | 52 | Chitinase family 18 protein | YP_169730 |
| FTT0721c | 82.4, 5.37 | 5737 | 62 | Peroxidase/catalase | YP_169735 |
| FTT0863c | 22.0, 5.63 | 360 | 36 | LemA-like protein | *YP_169865* |
| FTT1060c | 16.1, 5.55 | 147 | 39 | 50S ribosomal protein L9 | YP_170041 |
| FTT1103 | 38.7, 5.23 | 562 | 45 | Conserved hypothetical lipoprotein | *YP_170079* |
| FTT1269c | 69.2, 4.88 | 953 | 43 | Chaperone protein dnaK | YP_170225 |
| FTT1303c | 33.1, 5.18 | 356 | 53 | Hypothetical protein | YP_170257 |
| FTT1358c | 58.8, 4.69 | 76 | 4 | Intracellular growth locus,subunit B | *YP_170310* |
| FTT1373 | 35.2, 5.41 | 169 | 19 | 3-oxoacyl-[acyl carrier protein] | YP_170322 |
| FTT1374 | 33.5, 5.08 | 2771 | 80 | Malonyl coA-acyl carrier protein | YP_170323 |
| FTT1389 | 28.7, 5.55 | 86 | 8 | oxobutanoatehydroxymethyltransferase | YP_170334 |
| FTT1441 | 18.5, 5.34 | 155 | 26 | Hypothetical protein | *YP_170379* |
| FTT1484c | 67.2, 4.77 | 2051 | 46 | Pyruvate dehydrogenase, E2 component | YP_170419 |
| FTT1530 | 100.5, 5.51 | 186 | 24 | Fusion product of 3-hydroxacyl-CoA | YP_170460 |
| FTT1540c | 22.4, 9.28 | 100 | 16 | Hypothetical protein | *YP_170468* |
| FTT1696 | 57.4, 4.96 | 8992 | 68 | Chaperone protein, groEL | YP_170601 |
| FTT1768c | 65.7, 4.57 | 122 | 11 | Chitinase | YP_170659 |
| FTT1769c | 95.9, 5.45 | 523 | 27 | ClpB protein | YP_170660 |
| FTT1778c | 13.7, 8.67 | 85 | 38 | Hypothetical membrane protein | *YP_170667.1* |

**Supplementary Table S1 1 *nLCMS/MS identification of immunoreactive proteins from tryptic digests*.**

^a^ Locus tag also refers to annotated 2DE gels and immunoblots in Fig. 1 and Fig. 2.
^b^ Predicted molecular mass and isoelectric point of identified protein obtained from translated open reading frames of the *F. tularensis* SchuS4 genome sequence.
^c^ Cumulative Mascot score and ^d^ % sequence coverage of all tryptic peptides identified by nLC–MS/MS. A Mascot score of over 25 was required for positive identification. All MS/MS spectra were verified manually.

^e^ Accession number of protein homologue in *F. tularensis* SchuS4, matching tryptic peptides from excised spot, identified by nLC–MS/MS. Accession number for LVS protein listed in brackets.
